# Carnosic Acid and Its Semisynthetic Derivatives as Promising Anticancer Agents

**DOI:** 10.3390/ijms27031149

**Published:** 2026-01-23

**Authors:** Sara P. S. P. Moura, Vânia M. Moreira, Jorge A. R. Salvador

**Affiliations:** 1 Laboratory of Pharmaceutical Chemistry, Faculty of Pharmacy, University of Coimbra, 3000-548 Coimbra, Portugal; spmoura17@gmail.com (S.P.S.P.M.); vmoreira@ff.uc.pt (V.M.M.); 2 Center for Neuroscience and Cell Biology (CNC), University of Coimbra, 3004-504 Coimbra, Portugal; 3 Centre for Innovative Biomedicine and Biotechnology (CIBB), University of Coimbra, 3004-504 Coimbra, Portugal; 4 Drug Research Program, Division of Pharmaceutical Chemistry and Technology, Faculty of Pharmacy, University of Helsinki, 00014 Helsinki, Finland

**Keywords:** natural products, abietane, diterpenoids, carnosic acid, semisynthetic derivatives, anticancer activity

## Abstract

Natural products are a valuable source of structurally diverse bioactive compounds, many of which have contributed significantly to the discovery of new anticancer drugs. Carnosic acid **1**, an abietane-type diterpenoid primarily found in rosemary and sage, has emerged as a promising scaffold due to its ability to modulate key cellular pathways involved in cancer development and progression, including the cell cycle, apoptosis, autophagy, inflammation, and oxidative stress. Despite its multifaceted approach to combat cancer and promising results obtained in vitro and in vivo, its moderate potency limits its clinical application. To address this limitation, several chemical modifications have been performed to generate semisynthetic derivatives with improved efficacy. Several semisynthetic derivatives have demonstrated significantly enhanced anticancer activity across diverse cancer models, highlighting the importance of structural optimization of the carnosic acid **1** backbone. This review provides a comprehensive overview of carnosic acid **1** and its semisynthetic derivatives, focusing on their anticancer activities, underlying molecular mechanisms, and structure–activity relationships, with the aim of guiding the future design and development of carnosic acid **1**-derived anticancer drugs.

## 1. Introduction

Cancer remains a major public health, societal, and economic issue in the 21st century and is one of the leading causes of morbidity and mortality worldwide [1]. According to the 2024 GLOBOCAN report, an estimated 20 million new cancer cases were diagnosed worldwide in 2022, leading to approximately 9.7 million deaths. Although cancer treatments have advanced, their effectiveness is still hindered by drug resistance and severe side effects [2,3,4]. These limitations highlight the need for new anticancer agents that are both more effective and selective for cancer cells.

Natural products offer a rich reservoir of structurally diverse scaffolds with broad activities, making them valuable tools in drug discovery for covering larger areas of chemical space [5,6,7,8,9,10,11]. Compared to fully synthetic compounds, some will exhibit uniquely complex chemical structures characterized by a higher proportion of sp^3^-hybridized carbons and stereocenters, ring systems, higher molecular masses, more H-bond donors and acceptors, and numerous oxygen atoms while containing fewer halogen and nitrogen atoms [7,12,13]. The presence of molecular descriptors such as fraction sp^3^ (Fsp^3^) carbons, and the number of stereochemical centers has been previously shown to correlate with clinical success [12,14]. In addition, the intrinsic molecular rigidity of some natural products has supported effective interactions with complex biological targets, including protein–protein interactions. Taken together, these properties distinguish natural products from synthetic scaffolds and highlight their importance in anticancer drug discovery.

Over the past few decades, extensive research has focused on the isolation of novel compounds and the evaluation of their anticancer properties [5,6,7,8,9,10,11]. Several of the most effective and clinically established anticancer drugs used in cancer therapy are natural products or natural-derived compounds. Notable examples include plant-derived agents, such as paclitaxel and its semisynthetic analogs docetaxel and cabazitaxel, as well as compounds from microorganisms and marine organisms, including mitomycin C and trabectedin [5,9,15,16]. This well-established clinical success provides a strong rationale for the continued exploration of natural products as sources of novel anticancer scaffolds. Despite their recognized potential, the clinical application of natural products is often limited by challenges such as low efficacy, unfavorable pharmacokinetic properties, high toxicity, and limited availability [17]. To overcome these limitations, chemical modification of scaffolds is often required to enhance these properties. Approximately 25% of the anticancer drugs approved between 1981 and 2019 were related to natural products, whereas only about 12% of the approved agents during this period were fully synthetic [6]. Notably, within the natural product-based group, only 30% were used in their natural form, whereas the majority (70%) were derived from natural products.

Within this context, carnosic acid (CA) **1** has emerged as a particularly interesting molecule. CA **1** is an abietane-type diterpenoid found mainly in *Rosmarinus officinalis* (rosemary) and *Salvia officinalis* (sage), both members of the Lamiaceae family [18,19]. Although the therapeutic use of sage and rosemary dates back to ancient times, the investigation of CA **1** properties and its mechanism of action as an isolated compound began only in the early 2000s [20]. Since then, CA **1** has been extensively investigated for its diverse pharmacological properties, encompassing antioxidant, anti-inflammatory, neuroprotective, cardioprotective, antiadipogenic, hepatoprotective, gastroprotective, antimicrobial, antidiabetic, and, notably, anticancer activities [18,20,21,22,23,24,25,26,27,28,29,30,31,32,33]. Accumulating evidence suggests that CA **1** exerts its anticancer effects, both in vitro and in vivo, through multiple mechanisms involving the modulation of the cell cycle, autophagy, apoptosis, oxidative stress, and inflammatory signaling pathways [18,20,21,22,31,32]. Although CA **1** exhibits promising anticancer activity, its moderate potency and selectivity limit its therapeutic potential. To overcome these limitations, chemical modification of the CA **1** has been explored as a strategy to enhance its efficacy [34,35,36,37,38,39,40,41]. Several semisynthetic derivatives of CA **1** have been synthesized and evaluated for their anticancer activities.

This review provides an integrated overview of the chemical structure, natural occurrence, and biosynthesis of CA **1**, as well as the anticancer activity of CA **1** and its semisynthetic derivatives. It highlights the molecular mechanisms underlying their anticancer activity, explores structure–activity relationships (SARs), and discusses their potential for the development of novel anticancer agents. To the best of our knowledge, this is the first review specifically focused on both the anticancer activity of CA **1** and its semisynthetic derivatives.

## 2. Chemical Structure and Natural Occurrence of Carnosic Acid

**Figure 1:**
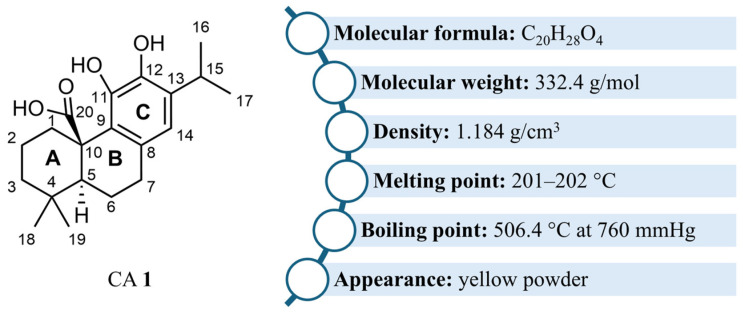
Structure and physicochemical properties of CA **1**.

CA **1** is a tricyclic diterpenoid with a planar abietane skeleton, featuring two six-membered rings (A and B rings) fused onto a third ring C which is a catechol (Figure 1) [18,19,21,42]. The two cyclohexane rings A and B form a *trans* ring junction, generating stereogenic centers at C5 and C10 [42,43,44]. A carboxyl group is present at C20 [18,19,21,42]. CA **1** is often also classified as a polyphenol owing to the presence of the catechol in its structure [22]. The physicochemical properties of CA **1** are presented in Figure 1.

CA **1** was first discovered in the leaves of sage (*Salvia officinalis*) in 1964 and later found in higher concentrations in the leaves of rosemary (*Rosmarinus officinalis*) in 1965 [18,19,20]. CA **1** has also been found in other *Salvia* species and genera within the Lamiaceae family [19]. However, *R. officinalis* is considered the richest natural source of CA **1**, with concentrations ranging from 3 to 50 mg/g of dry weight. To enhance the CA **1** content, several high-yielding rosemary cultivars have been developed, reaching concentrations between 4% and 10% of the weight of air-dried leaves.

Environmental conditions play a crucial role in modulating CA **1** abundance in plants. Studies have demonstrated that rosemary cultivated in cooler and more humid climates, such as those found in England, produces higher levels of CA **1**, compared to plants grown in warmer, arid environments typical of the Mediterranean regions. In addition, nutrient availability significantly influences CA **1** biosynthesis: elevated sodium levels have been shown to reduce its abundance in *Salvia officinalis*, whereas supplementation with potassium and calcium promotes its accumulation in the leaves of this species [19].

The catechol structure of CA **1** confers potent antioxidant properties, enabling the compound to neutralize reactive oxygen species (ROS) through oxidation to form carnosol and other related derivatives [19,20]. This oxidation process contributes to the defense mechanisms of plants against environmental stressors, such as ultraviolet (UV) radiation and drought.

## 3. Biosynthesis of Carnosic Acid

**Scheme 1:**
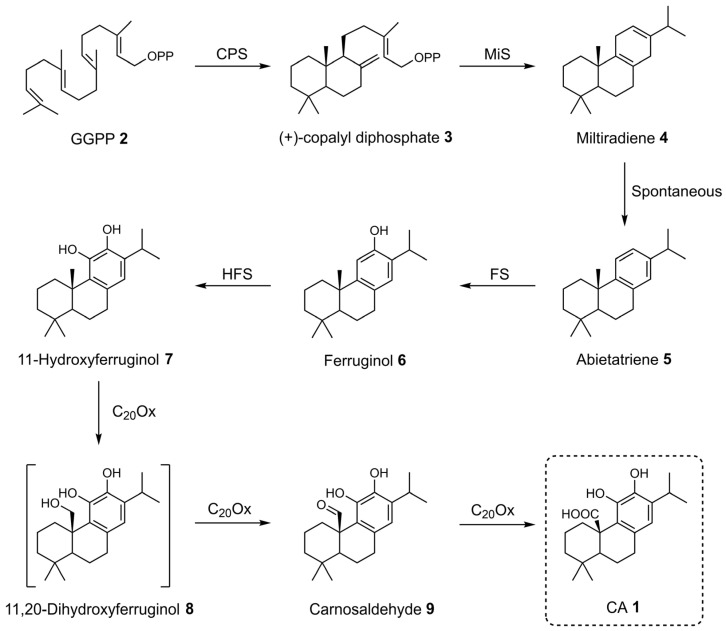
Proposed biosynthetic pathway of CA **1**.

CA **1** biosynthesis begins with the universal diterpenoid precursor geranylgeranyl diphosphate (GGPP) **2** [19,45,46,47]. The first step in this pathway involves the cyclization of GGPP **2** into (+)-copalyl diphosphate **3**, a reaction catalyzed by copalyl diphosphate synthases (CPS), which belong to class II diterpene synthases (Scheme 1). These enzymes initiate cyclization through the protonation of the carbon–carbon double bond. Subsequently, (+)-copalyl diphosphate **3** undergoes ionization of its diphosphate group, contributing to the continuation of the cyclization process and resulting in the formation of miltiradiene **4**. This last reaction is catalyzed by miltiradiene synthase (MiS), a class I diterpene synthase. Miltiradiene **4** spontaneously undergoes aromatization to form abietatriene **5**, although this process can be significantly accelerated by exposure to UV radiation. A crucial intermediate in the biosynthetic pathway is ferruginol **6**, which is formed through hydroxylation of abietatriene **5** at the C12 position. This transformation is catalyzed by cytochrome P450 monooxygenases (CYPs) of the CYP76 family, referred to as ferruginol synthases (FSs). This group of enzymes includes CYP76AH4 from *R. officinalis* and CYP76AH24 from *Salvia pomifera*, as well as other CYPs from *S. miltiorrhiza* and *S. fruticose* [45,47]. Further hydroxylation of ferruginol **6** at the C11 position produced 11-hydroxyferruginol **7**. The enzymes responsible for this reaction have been designed 11-hydroxyferruginol synthases (HFSs) by Scheler et al., who noted that they differ by three amino acids from FSs in *S. miltiorrhiza* [45]. The final step in the biosynthesis of CA **1** involves the oxidation of 11-hydroxyferruginol **7** at the C20 position, catalyzed by CYP76AK subfamily enzymes (notably CYP76AK6–8) from *R. officinalis*, *S. fruticosa*, and *S. pomifera* [45,47]. These enzymes exhibit C_20_ oxidase activity and are therefore referred to as C_20_Ox. The conversion is thought to occur via three sequential oxidation steps, as proposed by Scheler et al. [45].

## 4. Anticancer Activity of Carnosic Acid

The anticancer properties of CA **1** have been extensively investigated both in vitro and in vivo. A growing body of evidence demonstrates that CA **1** inhibits the proliferation of a wide range of cancer cell types, including brain, breast, cervical, colorectal, esophageal, gastric, leukemia, liver, lung, neuroblastoma, oral, ovarian, prostate, renal, and melanoma. The antiproliferative activity of CA **1** across these models, including the corresponding IC_50_ values and experimental conditions (exposure time and assay type), is summarized in Table 1. Furthermore, numerous studies have elucidated the molecular targets and signaling pathways modulated by CA **1**, as outlined in Table 2, Table 3, Table 4 and Table 5. The following discussion provides a comprehensive overview of the antiproliferative effects of CA **1** in different types of cancer, emphasizing the underlying molecular mechanisms.

CA **1** exhibited antiproliferative effects in PT2 glioblastoma cells, with an IC_50_ of 27.5 µM, exerting its effects by inducing apoptosis and arresting the cell cycle at the early G2 phase (Table 1 and Table 2) [48]. These effects are associated with proteasome-dependent degradation of regulatory proteins such as cyclin B1, SOX2, retinoblastoma (Rb), and glial fibrillary acidic protein (GFAP). Additionally, the expression of p21^WAF^ was upregulated, leading to CDK inhibition and cell cycle blockade. A concern in this study was the lack of selectivity of CA **1**, as it reduced the viability of both glioblastoma cells and normal human astrocytes.

CA **1** demonstrates a broad spectrum of activity across different breast cancer subtypes, including human epidermal growth factor receptor 2 (HER2)-positive and hormone receptor (HR)-positive subtypes as well as triple-negative breast cancer (Table 2) [49,67]. CA **1** inhibited cell proliferation, promoted apoptosis, and caused cell cycle arrest at the G2/M phase in 184-B5/HER cells, a model that represents an aggressive subtype of breast cancer [67]. This arrest was further supported by the upregulation of cyclin B1, a marker specific to the G2 phase. CA **1** also showed antiproliferative activity against HR-positive MCF-7 and triple-negative MDA-MB-231 cells by inducing apoptosis and oxidative stress [49]. It was more potent against MCF-7 cells than MDA-MB-231 cells, with IC_50_ values of 29.3 µM and 47.5 µM, respectively (Table 1).

CA **1** demonstrated significant anticancer activity against cervical cancer cells by inducing G2/M phase cell cycle arrest and promoting apoptosis (Table 2) [68]. It activates key mitogen-activated protein kinase (MAPK) pathways, including c-Jun N-terminal kinase (JNK), extracellular signal-regulated kinase 1/2 (ERK1/2), and p38, which are involved in the induction of apoptosis. Additionally, CA **1** increased ROS levels, leading to JNK phosphorylation, endoplasmic reticulum stress, and caspase-3 activation, which drives apoptosis. In vivo studies confirmed that administering 20 and 30 mg/kg of CA **1** for five weeks effectively inhibited tumor growth and progression in xenograft models, using the same mechanisms observed in vitro. CA **1** also shows promise in overcoming multidrug resistance in cervical cancer, particularly in the multidrug-resistant KB-C2 cell line that overexpresses P-glycoprotein (P-gp) [69]. It reverses P-gp-mediated drug resistance by inhibiting the efflux of chemotherapeutic agents, such as daunorubicin, thereby increasing their intracellular accumulation. Additionally, CA **1** stimulates P-gp ATPase activity and, therefore, enhances the cytotoxic effects of P-gp substrates, such as vinblastine, indicating its capacity to improve the efficacy of chemotherapy in multidrug-resistant cancer cells.

CA **1** demonstrates significant anticancer activity in colorectal cancer (CRC) through a variety of molecular and cellular mechanisms (Table 1 and Table 3) [51,52,53,70,71,72]. In human CRC cell lines, such as HT-29 and Caco-2, CA **1** induces cell cycle arrest at different phases, specifically the S-phase and G1-phase in HT-29 cells and the G2/M phase in Caco-2 cells [53,70,71]. The differences in the phase of cell cycle arrest observed in HT-29 cells may be attributed to variations in the experimental conditions, namely the concentration of CA **1** and duration of treatment. Specifically, S-phase arrest was induced after the incubation of cells with 1 and 10 µM of CA **1** for 48 h, while G1-phase arrest occurred when cells were treated with a higher concentration of 47.7 µM for 24 h [70,71]. CA **1** reduced the expression of cyclins and CDKs, which are essential for the transition between phases of the cell cycle [53,70]. It also targets E2F transcription factor 1 (E2F1)-regulated proteins involved in DNA replication, such as nucleosome assembly protein 1-like 1 (NAP1L1) and nuclear autoantigenic sperm protein (NASP), and proteins associated with DNA synthesis and cell cycle progression, including ribonucleotide reductase M1 (RRM1), DNA topoisomerase II alpha (TOP2A), COP9 signalosome complex subunit 8 (COPS8), and retinoblastoma-binding protein 4 (RBBP4) [71].

**Table 3:** In vitro anticancer activity of CA **1** against CRC.

Type of Cancer	Cell Lines	Effects on Targeted Cells	Molecular Pathways/Targets	Ref.
**Colorectal**	Caco-2	Antiproliferation Apoptosis Inhibition of cell adhesion, migration, and invasion	↓ uPA activity, MMP-2/9 activity, COX-2	[52]
		Antiproliferation Cell cycle arrest (G2/M phase)	↓ cyclin A	[53]
	HT-29	Antiproliferation Cell cycle arrest (G1 phase) Inhibition of cell adhesion, migration, and invasion	↑ GCNT3, HO-1, SQSTM1, HSPA5	[71]
			↓ RRM1, TOP2A, COPS8, RBBP4, GMPS, HPRT1, MKI67, NAP1L1, NASP	
		Antiproliferation Apoptosis	↑ ROS	[51]
			↓ p-JAK2, p-Src, survivin, cyclin D1/D2, p-STAT3	
		Antiproliferation Apoptosis Cell cycle arrest (S phase)	↑ Bax	[70]
			↓ Cyclin D1, CDK4, p-AKT, Bcl-xL	
	HCT116	Antiproliferation Apoptosis	↑ c-caspase-3, Nrf2, sestrin-2	[72]
		Antiproliferation Apoptosis	↑ p53, Bax, c-caspases-3/9, c-PARP, ROS	[51]
			↓ Mdm2, Bcl-2, Bcl-xl, p-JAK2, p-Src, survivin, cyclin D1/D2/D3, p-STAT3	
	SW480	Antiproliferation	↑ Nrf2, PERK	[72]
		Antiproliferation Apoptosis	↑ ROS	[51]
			↓ p-JAK2, p-Src, survivin, cyclin D1/D2, p-STAT3	

Another mechanism underlying the antiproliferative effects of CA **1** in CRC cells is the induction of apoptosis, characterized by the upregulation of pro-apoptotic proteins, such as Bcl-2-associated X protein (Bax) and p53, and downregulation of anti-apoptotic markers, including B-cell lymphoma 2 (Bcl-2), B-cell lymphoma extra large (Bcl-xL), and mouse double minute 2 (Mdm2) [51,52,70,72]. This apoptotic response was further confirmed by increased cleavage of caspases-3 and -9, as well as Poly(ADP-ribose) polymerase (PARP), indicating activation of the intrinsic apoptotic pathway [51,72].

Kim et al. observed that the central mechanism driving the anticancer effects of CA **1** was the generation of ROS in CRC cells [51]. ROS not only triggers apoptosis but also mediates the suppression of the signal transducer and activator of transcription 3 (STAT3) signaling pathway by inhibiting the upstream phosphorylation of Janus kinase 2 (JAK2) and Src, resulting in decreased STAT3 activation. Consequently, this led to the downregulation of STAT3 downstream targets, including survivin and cyclins D1, D2, and D3, thereby contributing to the observed antiproliferative effects.

CA **1** has demonstrated anticancer activity in CRC by targeting both tumor cells and the surrounding metabolic microenvironment that supports tumor growth [70]. In co-culture systems of HT-29 CRC cells with 3T3-L1 adipocytes, CA **1** effectively inhibited cancer cell growth by inducing cell cycle arrest and apoptosis. These effects were closely linked to the suppression of leptin receptor (Ob-R) signaling, as evidenced by the reduced phosphorylation of ERK and protein kinase B (AKT). In addition to its effects on cancer cells, CA **1** also impeded the differentiation of preadipocytes, as shown by a decrease in triglyceride accumulation. These findings were further supported by in vivo studies using the colitis-associated CRC model, in which CA **1** significantly reduced tumor formation, decreased circulating levels of pro-tumorigenic hormones (leptin, adiponectin, insulin, and IGF1), and downregulated key metabolic and oncogenic markers in colorectal tissue, including the insulin receptor, Ob-R, p-AKT, cyclin D, and Bcl-xL [70].

CA **1** also reduces the invasive capacity of CRC cells by inhibiting cell adhesion and migration, mechanisms mediated through the suppression of proteases such as matrix metalloproteinase (MMPs) and urokinase plasminogen activator (uPA), the upregulation of glucosaminyl (N-acetyl) transferase 3 (GCNT3), and the downregulation of cyclooxygenase-2 (COX-2) expression [52,71].

CA **1** exerts part of its anticancer activity in CRC cells through activation of the Nuclear factor erythroid 2-related factor 2 (Nrf2)-mediated oxidative stress response [72]. This activation is closely linked to the induction of endoplasmic reticulum stress via the phosphorylation of protein kinase R-like endoplasmic reticulum kinase (PERK). Phosphorylated PERK functions as a direct kinase for Nrf2, promoting its release from Kelch-like ECH- associated protein 1 (Keap1) and enabling its nuclear translocation, where it binds to antioxidant response elements (AREs), inducing the expression of cytoprotective genes. Among these, sestrin-2, a key regulator of oxidative stress and redox homeostasis, was significantly upregulated in HCT116 cells treated with CA **1**, suggesting its tumor-suppressive role. Notably, this effect appears to be specific to certain cell lines, as CA **1** did not activate the Nrf2/sestrin-2 axis in SW480 cells, suggesting differences in redox sensitivity or responsiveness to endoplasmic reticulum stress among different CRC subtypes.

In HT-29 cells, activation of the Nrf2 pathway by CA **1** was further evidenced by the increased expression of downstream targets, including heme oxygenase 1 (HO-1) and sequestosome-1 (SQSTM1) [71]. Proteomic analyses also confirmed that CA **1** induces endoplasmic reticulum stress in HT-29 cells, as indicated by the upregulation of unfolded protein response (UPR)-associated proteins, such as heat shock protein family A (Hsp70) member 5 (HSPA5).

Li et al. explored the anticancer potential of CA **1** in the adenomatous polyposis coli (Apc) ^Min/+^ mouse model of CRC, with particular emphasis on its anti-inflammatory effects and ability to modulate the gut microbiota [73]. Administration of 3 mg/kg of CA **1** over eight weeks significantly inhibited tumor growth and progression, reduced angiogenesis, and showed no adverse effects on other organs, highlighting its safety profile. CA **1** favorably altered the gut microbiota by increasing anti-inflammatory bacteria and reducing harmful bacteria, which was associated with changes in DL-lactic acid production. These microbiota shifts contributed to a reduced inflammatory response, as evidenced by decreased levels of interleukin (IL)-1β, IL-6, and IL-17A. The anti-inflammatory action of CA **1** ultimately led to the inhibition of the nuclear factor-kappa B (NF-κB)/STAT3 signaling pathway, contributing to its anticancer activity [73].

In esophageal squamous cell carcinoma, CA **1** demonstrated anticancer potential by inducing G2/M cell cycle arrest and promoting apoptosis through modulation of key regulatory proteins (Table 4) [74]. Furthermore, CA **1** also suppressed cell migration and invasion by inhibiting the MAPK signaling pathways, including ERK, p38, and JNK.

In gastric and lung cancer models, CA **1** has demonstrated promising antitumor activity through modulation of the phosphoinositide 3-kinase (PI3K)/AKT/mammalian target of rapamycin (mTOR) signaling cascade, a central pathway involved in cell proliferation and survival (Table 4) [55,60]. In gastric cancer cells, another key effect of CA **1** was the downregulation of survivin, an anti-apoptotic protein frequently overexpressed in tumors, leading to apoptosis [55]. In lung cancer cells, CA **1** promoted cell death through mechanisms that varied according to the subtype of non-small cell lung cancer (NSCLC) [59,60]. In A549 cells, CA **1** induced apoptosis and effectively suppressed migration and invasion, an effect associated with the downregulation of MMP-9 [60]. However, in H1299 cells, CA **1** triggered both apoptosis and autophagy, contributing to its antiproliferative effect [59]. This dual mechanism was linked to the activation of the sestrin-2/liver kinase B1 (LKB1)/5′ AMP-activated protein kinase (AMPK) signaling pathway. The induction of autophagy in H1299 cells was confirmed by the increased expression of LC3-II, a well-established autophagy marker.

**Table 4:** In vitro anticancer activity of CA **1** against esophageal, gastric, leukemia, liver, lung, and neuroblastoma cancers.

Type of Cancer	Cell Lines	Effects on Targeted Cells	Molecular Pathways/Targets	Ref.
**Esophageal**	KYSE-150	Antiproliferation Apoptosis Cell cycle arrest (G2/M phase) Inhibition of cell migration and invasion	↑ γ-H2AX, Bax, c-caspase-3	[74]
			↓ cyclin B1, Mdm2, CDK1, Bcl2, p-ERK, p-JNK, p-p38	
**Gastric**	AGS	Antiproliferation Apoptosis Cell cycle arrest (G1 phase)	↑ c-PARP, caspases-3/8/9 activity	[55]
			↓ survivin, p-mTOR, mTOR, p-AKT, AKT1	
**Leukemia**	KBM-7	Antiproliferation Apoptosis Cell cycle arrest (G2/M phase) Inhibition of cell invasion	↓ microRNA-780	[56]
	CCRF-CEM	Apoptosis	↑ p53, c-PARP	[57]
			↓ ROS, FAK	
	CEM/ADR5000	Apoptosis	↑ p53, c-PARP	[57]
			↓ FAK	
**Liver**	HepG2 SMMC-7721	Antiproliferation Apoptosis Cell cycle arrest (G2/M phase) Inhibition of cell migration	↑ Bax, Bad, ROS, c-caspases-3/8/9, c-PARP	[58]
			↓ Bcl-2, MMP	
	HepG2	Antiproliferation Autophagia	↑ LC3-II	[75]
			↓ p-AKT, p-mTOR	
**Lung**	A549	Antiproliferation Apoptosis Inhibition of cell migration and invasion	↓ MMP-9, p-mTOR, p-PI3K, p-AKT	[60]
	H1299	Antiproliferation Apoptosis Autophagy	↑ c-caspases-3/7, c-PARP, Bax, sestrin-2, p-LKB1, p-AMPK, nuclear condensation, LC3-II	[59]
			↓ Bcl-2	
**Neuroblastoma**	IMR-2	Antiproliferation Apoptosis	↑ c-caspases-3/9, c-PARP, ROS, p-p38	[61]
			↓ Bcl-2, p-ERK, p-JNK1	

Mahmoud et al. investigated the anticancer activity of CA **1** across a panel of National Cancer Institute (NCI) tumor cell lines, including brain, breast, colorectal, kidney, leukemia, lung, melanoma, ovary, and prostate [57]. They found variable sensitivity among cancer types, with leukemia cells being the most responsive to CA **1**, whereas ovarian and colorectal carcinomas showed greater resistance. Mechanistic studies showed that CA **1** induced apoptosis in both multidrug-resistant leukemia cells (CEM/ADR5000) and their drug-sensitive counterparts (CCRF-CEM), potentially through modulation of focal adhesion kinase (FAK) (Table 4). Notably, CA **1** exhibited greater efficacy against resistant cells (CEM/ADR5000), with an IC_50_ of 11.48 µM, compared to the drug-sensitive cells (CCRF-CEM), with an IC_50_ of 19.50 µM (Table 1). Another study demonstrated that CA **1** inhibited the proliferation and invasion of KBM-7 leukemia cells by suppressing microRNA-780, a molecule implicated in cancer progression [56]. Notably, CA **1** demonstrated exceptional cytotoxic activity against the HL-60 cell line, with an IC_50_ value of 1.7 µM after 24 h of exposure (Table 1) [54].

CA **1** demonstrated anticancer activity in liver cancer cells, triggering distinct mechanisms of cell death (Table 4) [58,75]. In human hepatoma HepG2 cells, CA **1** induced autophagic cell death through inhibition of the AKT/mTOR signaling pathway, as evidenced by an increase in the LC3-II/LC3-I ratio and the formation of autophagic vacuoles [75]. Additionally, CA **1** can induce cell death via the ROS-mediated intrinsic apoptotic pathway in HepG2 cells, a mechanism also observed in SMMC-7721 cells [58]. CA **1** further induced G2/M cell cycle arrest and inhibited cell migration in both cell types. In vivo, treatment with 20 mg/kg CA **1** reduced liver tumor growth in HepG2 and SMMC-7721 xenograft mouse models without affecting body weight or liver function, indicating a favorable safety profile. In vivo studies further supported the in vitro findings, showing that CA **1** modulates the expression of apoptosis-related proteins. Moreover, CA **1** reduced the phosphorylation of mTOR and NF-κB while increasing the expression of IL-2 within tumor tissues, indicating its potential as an effective anticancer agent for liver cancer [58].

CA **1** inhibits the proliferation of IMR-32 neuroblastoma cells (IC_50_ = 30 µM) by inducing apoptosis through a mechanism involving oxidative stress (Table 1 and Table 4). CA **1** treatment increases the generation of ROS, while pretreatment with the antioxidant N-acetylcysteine (NAC) suppresses both ROS production and apoptosis, confirming the critical role of oxidative stress. CA **1** also activated the p38 and JNK MAPK pathways and reduced ERK activation. Importantly, NAC attenuated p38 phosphorylation, and silencing p38 decreased caspase-3 activation. These findings suggest that CA **1** promotes apoptosis in IMR-32 cells via an ROS-dependent mechanism mediated by p38 MAPK activation [61].

In oral squamous cell carcinoma (OSCC), CA **1** inhibited the viability of CAL27 and SCC9 cells, with IC_50_ values of 34.71 and 32.73 µM, respectively (Table 1) [62]. Notably, CA **1** reduced OSCC cell viability without affecting normal oral keratinocytes. Furthermore, CA **1** enhanced the susceptibility of cisplatin-resistant cells (CAL27-DDP and SCC9-DDP) to cisplatin by inducing ferroptosis, a type of programmed cell death (Table 5). This effect was achieved through the inhibition of the Nrf2/HO-1 signaling pathway and its downstream target, the cystine/glutamate antiporter solute carrier family 7 member 11 (xCT), a critical regulator of ferroptosis.

CA **1** exhibits antiproliferative activity against both androgen-sensitive (LNCaP) and androgen-refractory (PC-3, DU145) prostate cancer cell lines (Table 1 and Table 5) [63,64]. Importantly, CA **1** displayed selectivity toward cancer cells, as evidenced by its higher IC_50_ value (139.4 µM) in normal prostate epithelial cells (PNT1A), suggesting a favorable therapeutic window. In PC-3 cells, CA **1** inhibited the signaling axis formed by AKT, mammalian target of rapamycin complex 1 (mTORC1), and ribosomal protein S6 kinase beta-1 (p70S6K), while activating the sestrin-2/AMPK pathway, resulting in reduced cell viability [63]. Additionally, CA **1** induces both intrinsic and extrinsic apoptotic pathways in PC-3 cells [64]. The extrinsic pathway is demonstrated by the activation of caspase-8, whereas the intrinsic pathway is indicated by the activation of caspase-9. The cleavage of the BH3-interacting domain death agonist (Bid), which links the two apoptotic pathways, further supports this dual activation. This process is associated with increased activity of serine/threonine protein phosphatase 2A (PP2A), which suppresses the AKT/IκB kinase (IKK)/NF-κB signaling pathway, triggering a cascade of events that culminates in apoptosis. Similar pro-apoptotic effects were observed in DU145 cells, where CA **1** mainly activated the intrinsic pathway [64]. These findings suggest that CA **1** may be a promising candidate for targeting both hormone-sensitive and castration-resistant forms of prostate cancer.

**Table 5:** In vitro anticancer activity of CA **1** against oral, prostate, renal, and skin cancers.

Type of Cancer	Cell Lines	Effects on Targeted Cells	Molecular Pathways/Targets	Ref.
**Oral**	CAL27-DDP SCC9-DDP	Antiproliferation Ferroptosis	↑ ROS, lipid peroxidation levels	[62]
			↓ GSH levels, Nrf2, HO-1, xCT	
**Prostate**	PC-3	Antiproliferation	↑ p-AMPK, p-ACC, sestrin-2	[63]
			↓ p-AKT, p-mTOR, p-p70S6K	
		Antiproliferation Apoptosis	↑ caspases-3/7/8/9, c-PARP, PP2A activity, Bax, cytosol cytochrome c	[64]
			↓ XIAP, cIAP1, cIAP2, IKK activity, p-IkBα, Bid, Bcl-2, p-AKT, nuclear NF-*κ*B/p50, nuclear NF-*κ*B/p65	
	DU145	Antiproliferation Apoptosis	↑ caspases-3/7/9, c-PARP, Bax, cytosol cytochrome c	[64]
			↓ Bcl-2	
**Renal**	Caki	Antiproliferation Apoptosis	↑ c-caspase 3/7/8/9, c-PARP, Bax, cytoplasm cytochrome c, p53, p27, ROS, DR4, DR5	[76]
			↓ Bcl-2, Bcl-xL, Mdm2, p-STAT3, c-Myc, p-Src, cyclin D1/D2/D3, survivin	
**Skin**	B16F10	Antiproliferation Cell cycle arrest (G0/G1)	↑ p21	[66]
			↓ p27	
		Inhibition of cell migration and invasion	↑ TIMP-2, E-cadherin	[77]
			↓ MMP-9, TIMP-1, uPA, VCAM-1, Snail, Slug, vimentin, N-cadherin, p-Src, p-FAK, p-AKT	
	A375	Antiproliferation Apoptosis Cell cycle arrest (G2/M phases)	N.D.	[65]

N.D.: not determined.

In Caki renal carcinoma cells, CA **1** induces apoptosis via both intrinsic and extrinsic pathways [76]. The intrinsic pathway is activated through CA **1**-induced ROS generation, which leads to the activation of the tumor suppressor p53. This, in turn, triggers mitochondrial dysfunction, promoting cytochrome c release and caspase-9 activation. In the extrinsic pathway, CA **1** enhances the expression of death receptors 4 and 5 (DR4 and DR5), and activates caspase-8, which are hallmarks of this apoptotic pathway. Furthermore, CA **1** suppressed Src phosphorylation, leading to a reduction in STAT3 activation. As a result, downstream STAT3 target genes involved in proliferation and survival, such as D-type cyclins, survivin, and c-Myc, are downregulated, contributing to the overall antiproliferative and pro-apoptotic effects of CA **1** in renal carcinoma cells [76].

CA **1** induces cell cycle arrest at distinct phases depending on the melanoma cell line [65,66]. In B16F10 cells, CA **1** exerted its antiproliferative effects (IC_50_ = 7.08 µM) by inducing cell cycle arrest at the G0/G1 phase, which was supported by the upregulation of p21 expression [66]. In A375 cells, CA **1** arrested the cell cycle in the G2/M phase, which is consistent with the microtubule disruption observed in these cells [65]. CA **1** exhibits anti-metastatic properties in B16F10 melanoma cells by impairing the key processes involved in cell migration and invasion [77]. These effects were linked to decreased secretion of MMP-9, tissue inhibitor of metalloproteinases-1 (TIMP-1), urokinase plasminogen activator (uPA), and vascular cell adhesion molecule 1 (VCAM-1), along with an increase in tissue inhibitor of metalloproteinases-2 (TIMP-2) levels, which disrupted extracellular matrix remodeling. CA **1** also inhibited epithelial–mesenchymal transition (EMT), as evidenced by the downregulation of mesenchymal markers (zinc finger protein SNAI2 (Slug), zinc finger protein SNAI1 (Snail), N-cadherin, vimentin) and upregulation of the epithelial marker E-cadherin. Mechanistically, CA **1** suppressed the phosphorylation of FAK, Src, and AKT, key regulators of EMT and metastatic signaling. In vivo, the daily administration of CA **1** (50 mg/kg) for 7 days inhibited tumor growth in B16F10-xenograft C57BL/6 mice [66]. Treatment with CA **1** did not significantly alter the serum levels of aspartate aminotransferase, alanine aminotransferase, total bilirubin, or albumin, indicating no evidence of hepatotoxicity. These findings suggest that CA **1** exhibits antitumor activity without compromising liver function in treated mice.

**Figure 2:**
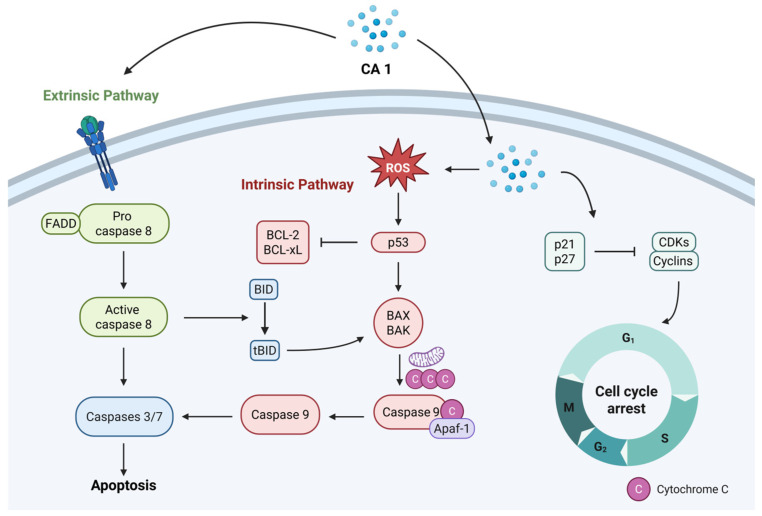
Schematic representation of the main effects induced by CA **1**, namely apoptosis and cell cycle arrest, which underlie its anticancer activity.

**Figure 3:**
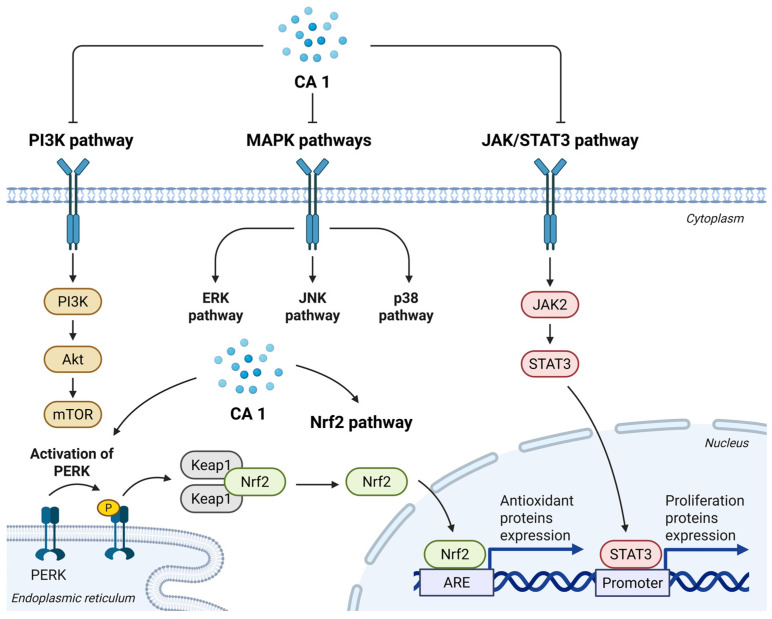
Schematic overview of the main signaling pathways modulated by CA **1** that contribute to its anticancer effects.

Overall, CA **1** exerts its antiproliferative effects mainly through the induction of apoptosis and cell cycle arrest, leading to the sustained inhibition of tumor cell proliferation (Figure 2). CA **1** modulates several signaling pathways that control proliferation, survival, stress responses, migration, and invasion, as summarized in Figure 3.

## 5. Semisynthetic Derivatives of Carnosic Acid

Although CA **1** exhibited promising anticancer activity, the biological data presented in the previous section indicated that its antiproliferative effects are generally achieved at relatively high micromolar concentrations and display considerable variability across different cancer models, which limits its therapeutic potential. To overcome these limitations, chemical modifications of the molecule have been explored as a strategy to enhance its efficacy.

In the reviewed studies, CA **1** was obtained through extraction and isolation from plants or from commercial sources [35,36,37,38,39,40,41]. When isolated from natural sources, CA **1** was most commonly obtained from the aerial parts of *Rosmarinus officinalis*, although its isolation from the aerial parts of *Perovskia abrotanoides* has also been reported [35,36,37,38]. Alternatively, the use of commercially available CA **1** can simplify the synthetic workflow by bypassing the extraction and isolation steps, thereby reducing the overall time required for the preparation of derivatives, and may represent a cost-effective option due to its competitive pricing. Overall, the preparation of semisynthetic CA **1** derivatives was relatively straightforward and did not require expensive reagents. While reaction times and yields vary depending on the type of chemical modification and purification steps, most derivatives can be obtained within reasonable time frames and with moderate to good yields. Several semisynthetic derivatives of CA **1** have been synthesized and evaluated for their anticancer activity. These modifications focused on the carboxylic acid at position C20, hydroxyl groups at positions C11 and C12, and the benzylic position (C7). In this section, the main semisynthetic derivatives reported to date are presented, with a particular focus on their structural features and biological activity.

**Figure 4:**
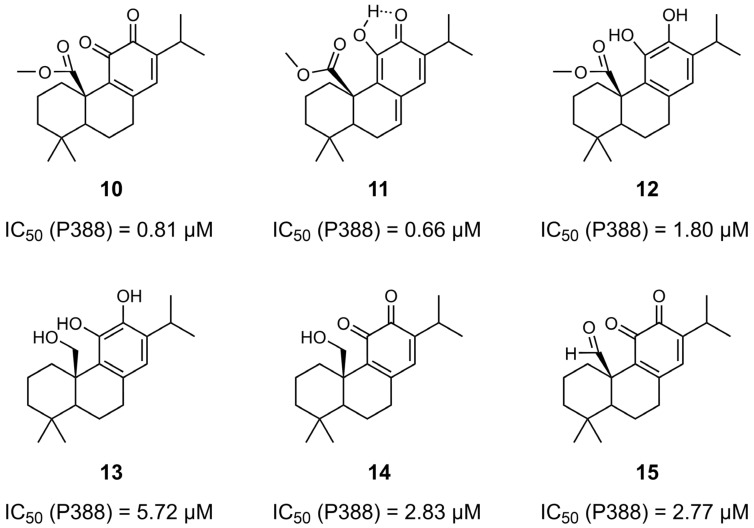
Chemical structures of derivatives **10**–**15** of CA **1** and their corresponding IC_50_ values determined in P388 murine leukemia cells. IC_50_ values were obtained using the MTT assay after 48 h of incubation with increasing concentrations of each compound [35].

Aoyagi et al. synthesized a series of CA **1** derivatives (**10**–**15**) by introducing structural modifications at the C20 position and at the C-ring (Figure 4) [35]. These compounds were evaluated for their antiproliferative activity against P388 murine leukemia cells. Among them, derivatives **10** and **11**, which feature both an ester group at C20 and an *o*-quinone or *o*-quinone equivalent structure in the C-ring, exhibited the highest activity, with IC_50_ values of 0.81 µM and 0.66 µM, respectively. The results obtained were significantly superior to those observed for CA **1** (IC_50_ = 3.61 µM). Notably, the presence of *o*-quinone (or its equivalent) in the C-ring appears to be crucial for antiproliferative activity, as evidenced by the lower activity of derivative **12** (IC_50_ = 1.80 µM), whose only structural difference relative to the other two derivatives is the absence of this feature. A similar trend was observed with derivatives **13** and **14**, where the introduction of an *o*-quinone moiety in compound **14** led to a marked improvement in activity, reducing the IC_50_ from 5.72 µM (derivative **13**) to 2.83 µM (derivative **14**).

**Figure 5:**
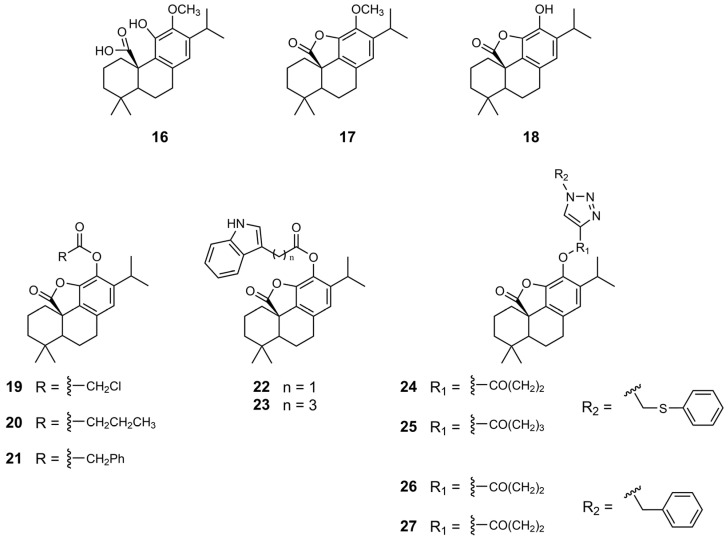
Chemical structures of derivatives **16**–**27** of CA **1**.

In 2010, Pertino et al. synthesized a panel of ester derivatives of CA **1** γ-lactone by incorporating aliphatic, aromatic, and heterocyclic substituents at the C12 position of the backbone (Figure 5) [36]. They also explored the effect of methyl ether at the same position. The derivatives were evaluated for their antiproliferative activity against gastric (AGS) and liver (HepG2) cancer cell lines (Table 6). To assess selectivity, the compounds were also tested on normal lung fibroblasts (MRC-5). The methylation of CA **1** at the C12 position to form compound **16** enhanced its antiproliferative activity in AGS cells, reducing the IC_50_ from 89.8 to 45.2 µM. Structural modification of compound **16** through the introduction of a γ-lactone ring, resulting in compound **17**, led to a significant increase in potency in gastric cells (IC_50_ = 26.7 µM). In contrast, the presence of the hydroxyl group at C12 on the derivative γ-lactone (compound **18**) markedly reduced the activity (IC_50_ > 200 µM). The introduction of different esters at the C12 position of CA **1** γ-lactone (derivatives **19**–**23**) generally enhanced the antiproliferative effects relative to derivative γ-lactone **18**. Notably, butyrate ester (compound **20**) improved efficacy in HepG2 cells (IC_50_ = 56.1 µM), and compound **23**, containing a C4 side chain linked to a heterocycle, was most effective in AGS cells (IC_50_ = 46.8 µM). Compound **23** was approximately twice as potent as CA **1** in AGS cells. Overall, the derivatives exhibited low selectivity toward cancer cells, as their IC_50_ values were similar in fibroblasts (MRC-5). For instance, derivative **17** had an IC_50_ of 31.9 µM in MRC-5 cells versus 26.7 µM in AGS cells; derivative **20** showed an IC_50_ of 44.4 µM in MRC-5 versus 56.1 µM in HepG2 cells; and derivative **23** displayed an IC_50_ of 54.5 µM in MRC-5 compared to 46.8 µM in AGS cells [36].

In 2015, Pertino et al. extended their research on the synthesis of ester derivatives of CA **1** γ-lactone (derivatives **24**–**27**), focusing on the introduction of substituted triazole, along with variations in the length of the linker between the core scaffold and triazole (Figure 5) [37]. The derivatives were more potent in gastric cancer cells (AGS cells) than in lung cancer cells (SK-MES-1) (Table 6). Compounds **24** and **25**, which differ in the number of methylene groups in the linker and contain a methyl phenyl sulfide substituent on the triazole moiety, exhibited similar antiproliferative activity. However, in derivatives **26** and **27**, the length of the linker influenced the antiproliferative activity, revealing a marked decrease in activity with increasing linker length. Derivative **26**, containing two methylene groups in the linker, was more active against both AGS and SK-MES-1 cells than derivative **27**, which contained three methylene groups. Nonetheless, these derivatives exhibit limited selectivity toward cancer cells [37].

**Figure 6:**
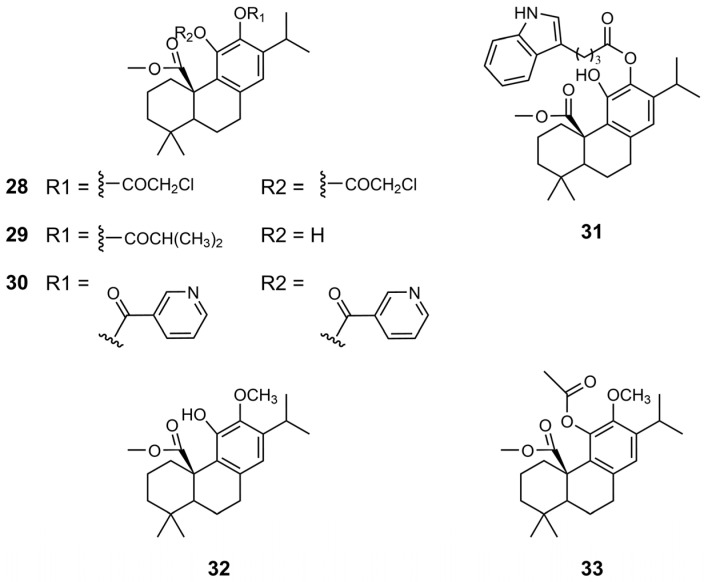
Chemical structures of derivatives **28**–**33** of CA **1**.

The same research team further investigated chemical modifications of CA **1** by synthesizing novel esters and ethers (derivatives **28**–**33**) (Figure 6) [38]. Esterification of the hydroxyl groups at the C11 and C12 positions using chloroacetic acid, along with the incorporation of an ester group at the C20 position, resulted in derivative **28**, which contributed to a marked enhancement of the antiproliferative activity in gastric cancer cells (Table 7). Derivative **28** exhibited the best result in AGS cells (IC_50_ of 17.7 µM), approximately fivefold more potent than CA **1**. In HepG2 cells, derivative **30**, featuring a nicotinate moiety, achieved the best results, with an IC_50_ of 23.3 µM. In general, the derivatives exhibited problems in selectivity. However, derivative **30** displayed a favorable balance in antiproliferative activity against cancer cells and normal cells.

**Figure 7:**
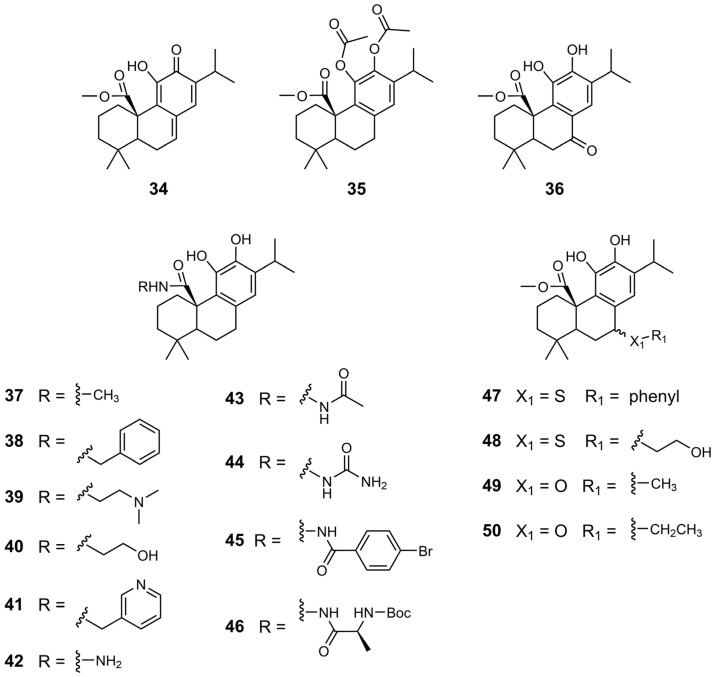
Chemical structures of derivatives **34**–**50** of CA **1**.

**Figure 8:**
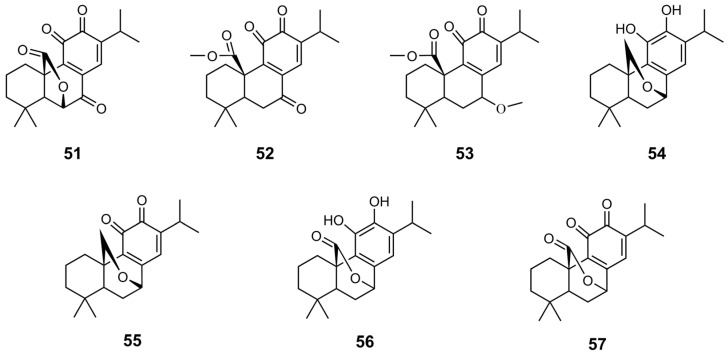
Chemical structures of derivatives **51**–**57** of CA **1**.

**Figure 9:**
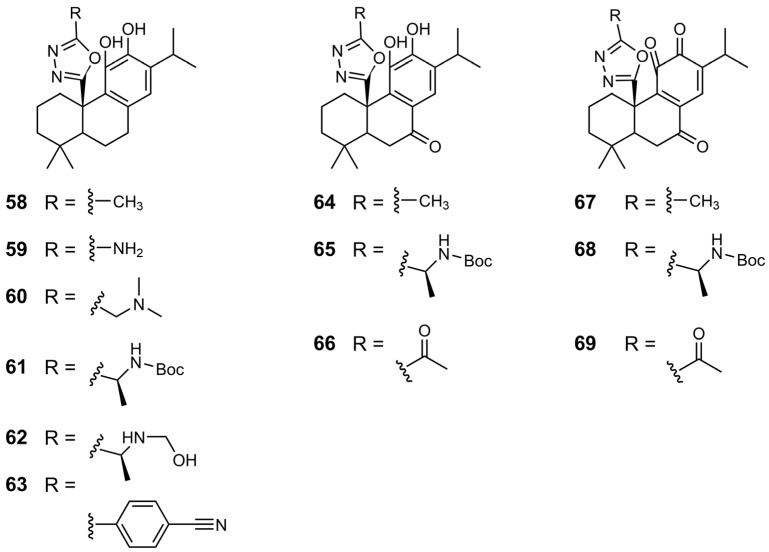
Chemical structures of derivatives **58**–**69** of CA **1**.

In 2019, Han et al. synthesized novel non-bisphosphonate inhibitors of human farnesyl pyrophosphate synthase (HsFPPS) using CA **1** as the lead compound [39]. HsFPPS is a key enzyme in the isoprenoid biosynthetic pathway, catalyzing the formation of farnesyl pyrophosphate, a crucial intermediate involved in protein prenylation, such as Ras proteins, and in the biosynthesis of cholesterol, dolichols, and coenzyme Q10. Inhibition of HsFPPS can downregulate the activity of oncogenic Ras, which is mutated in approximately 33% of all cancers. Bisphosphonates, such as zoledronate, effectively inhibit HsFPPS, but their poor cell permeability and tissue distribution reduce their therapeutic potential in oncology. As a result, there is growing interest in the development of alternative non-bisphosphonate HsFPPS inhibitors with improved pharmacological profiles. For this reason, Han et al. developed a panel of novel derivatives based on CA **1** as the lead compound, incorporating three main structural modifications: (1) modifications at the C20 position; (2) modifications at the benzyl position (C7); and (3) oxidation of hydroxyl groups on the C-ring (Figure 7, Figure 8 and Figure 9). The inhibitory activity of these compounds against HsFPPS was assessed, and their potency was quantified as IC_50_ values (Table 8) [39].

Esterification at the C20 position significantly enhanced the inhibitory activity against HsFPPs, with **12** and **34** achieving IC_50_ values of 0.865 and 0.833, respectively, approximately 20 times more potent than that of CA **1** [39]. Dual acetylation at the C11 and C12 positions of **12**, or benzylic oxidation, leading to compounds **35** and **36**, respectively, resulted in a marked reduction in inhibitory activity.

Overall, the introduction of an amide group at the C20 position (derivatives **37**–**46**) improved the activity of the compounds against HsFPPS compared to CA **1** [39]. The incorporation of a dimethylaminoethyl side chain (compound **39**) and a hydroxyethyl side chain (compound **40**) significantly enhanced the inhibitory activity, with IC_50_ values of 1.20 and 0.914 µM, respectively. These values were significantly lower than those of CA **1**, which had an IC_50_ of 20 µM. The presence of a hydrazine moiety in compound **42** significantly reduced enzymatic inhibition, resulting in an IC_50_ of 35 µM. However, the substitution of this amino group with acetyl (derivative **43**) led to a marked improvement, with **43** reaching an IC_50_ of 1.5 µM. Notably, the most potent inhibition was observed with more hydrophobic substituents, as observed for compounds **45** and **46**, which exhibited IC_50_ values in the 500–800 nM range [39].

Han et al. synthesized derivatives of **12** with modifications at the C7 position using sulfur or oxygen linker atoms (derivatives **47**–**50**) [39]. The most promising results were obtained with derivatives bearing hydroxyethyl (**48**) and methyl (**49**) substituents, both of which exhibited IC_50_ values of approximately 1 µM.

In general, the conversion of the catechol group to a quinone structure enhances the inhibitory activity of compounds [39]. Compounds **51** and **52** exhibited strong HsFPPS inhibition, with IC_50_ values of 0.389 and 0.523 µM, respectively. Oxidation of **49** produced compound **53**, which showed an IC_50_ of 0.234 µM, nearly 100 times more potent than CA **1**, and comparable to zoledronate. Similarly, oxidation of the hydroxyl groups of **54** and **56** produced **55** and **57**, respectively, resulting in a significant increase in inhibitory activity, approximately 30-fold [39].

This research group also synthesized derivatives of CA **1** featuring a 1,3,4-oxadiazole at the C20 position, exploring different substituents on the side chain of oxadiazole (derivatives **58**–**63**) [39]. Almost all derivatives demonstrated improved activity compared to that of CA **1**, with the most potent being **62**, which exhibited an IC_50_ of 0.194 µM. This value falls within the range of values of the reference compound zoledronate (IC_50_ = 0.1–0.2 µM). The introduction of a phenyl substituent, as seen in **63**, led to reduced activity, possibly due to steric hindrance within the binding site. Oxidation at the benzylic position did not enhance HsFPPS inhibition, as reflected by the loss of activity in compounds **64**–**66**. Consistent with previous observations, the presence of a quinone group significantly improved activity. Compounds **67**–**69** exhibited excellent IC_50_ values of approximately 300 nM [39].

The compounds were evaluated against pancreatic cancer cells, specifically PANC-1 and MiaPaca-2 cell lines, which are characterized by high expression of mutated Ras (KRAS), to assess their antiproliferative activity (Table 9) [39]. In enzymatic assays, most of the tested compounds were less potent than zoledronate in inhibiting HsFPPS activity. However, this trend was not observed at the cellular level. In fact, several derivatives exhibited superior antiproliferative effects against PANC-1 and MiaPaca-2 cells compared to zoledronate, likely owing to improved cell penetration [39].

The most potent derivative in PANC-1 cells was **47**, with an EC_50_ of 2.7 µM [39]. This represents a significant improvement over CA **1**, which had an EC_50_ of 15.0 µM. Interestingly, compound **47** showed weak inhibition of HsFPPS in the enzymatic assay (IC_50_ = 28.0 µM). However, among all the derivatives, **47** was the most potent inhibitor of *Homo sapiens* geranylgeranyl pyrophosphate synthase (HsGGPPS), with an IC_50_ of 5.5 µM. Since HsGGPPS inhibition can impair pancreatic cancer cell growth by disrupting Ras-mediated processes, the antiproliferative activity of compound **47** is likely attributed to HsGGPPS inhibition rather than HsFPPS inhibition [39].

Another notable finding is that most quinone-containing compounds (**51**, **52**, **57**, **67**, and **69**) exhibited weak antiproliferative activity against PANC-1 and MiaPaca-2 cells, with EC_50_ values exceeding 30 µM [39]. Despite this, these derivatives demonstrated strong inhibition of HsFPPS in the enzymatic assay, with IC_50_ values below 1 µM. This discrepancy suggests that the quinone moieties may react with cellular components, such as glutathione, before reaching their target, thereby diminishing their effectiveness in a cellular context [39].

In MiaPaca-2 cells, only the derivative **63** (EC_50_ = 1.60 µM) demonstrated stronger antiproliferative activity than CA **1** (EC_50_ = 5.50 µM) [39].

Additional studies were performed to elucidate the mechanism underlying the antiproliferative effects of compound **39** on PANC-1 cells [39]. This compound was selected based on its good correlation between cellular activity and HsFPPS inhibition, displaying an EC_50_ of 5.10 µM in PANC-1 cells and an IC_50_ of 1.20 µM against HsFPPS. The mechanism of action involves the inhibition of HsFPPS, resulting in decreased levels of farnesyl pyrophosphate and geranylgeranyl pyrophosphate, which subsequently diminishes membrane-associated Ras and triggers apoptosis in PANC-1 cells [39].

**Figure 10:**
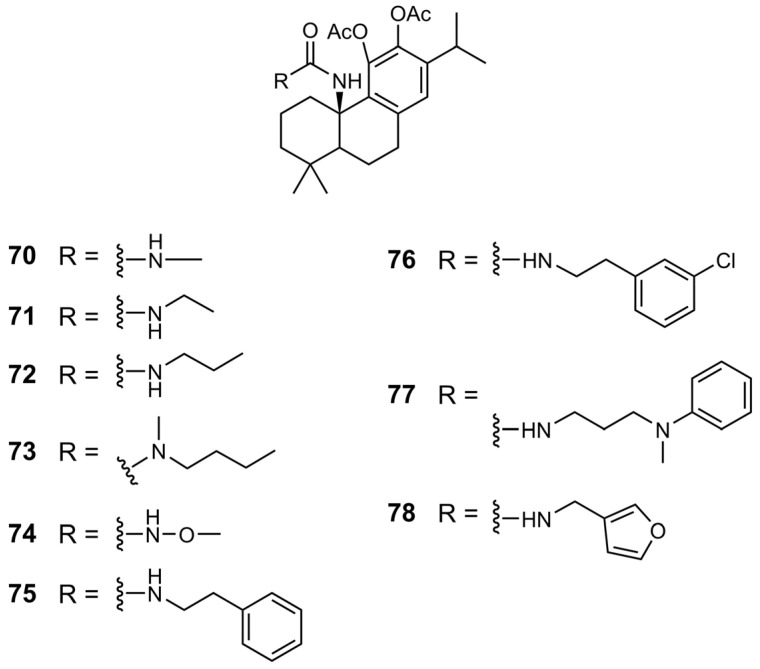
Chemical structures of derivatives **70**–**78** of CA **1**.

**Figure 11:**
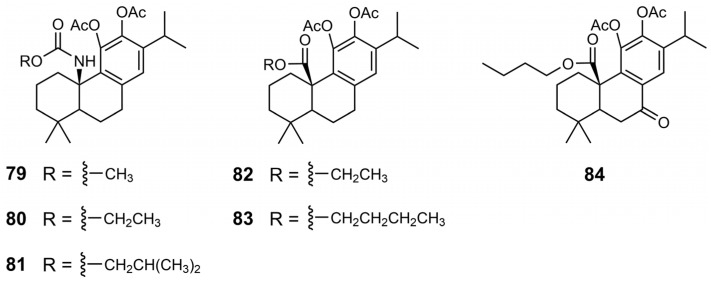
Chemical structures of derivatives **79**–**84** of CA **1**.

More recent studies have focused on the structural modifications of CA **1** by introducing urea, carbamate or ester groups at the C-20 position (Figure 10 and Figure 11) [40,41]. These modifications generally increased the antiproliferative activity of the derivatives relative to that of the parent compound in HCT116 CRC cells (Table 10).

In the urea and carbamate derivatives, an increase in the length of the aliphatic chain was associated with enhanced antiproliferative activity, resulting in lower IC_50_ values. For example, in the carbamate series, the IC_50_ values decreased from 55 µM for the derivative with a methyl chain (**79**) to 17 µM for the derivative bearing a 2-methyl propyl chain (**81**) [41]. In urea derivatives, the introduction of an electronegative atom also proved beneficial to activity, as demonstrated by the comparison between compounds **70** and **74**, with IC_50_ values of 55 and 41 µM, respectively [40]. A similar effect was observed for compounds **75** and **76**, where the incorporation of a chlorine atom on the aromatic ring led to increased potency, reducing the IC_50_ from 14 µM (derivative **75**) to 9.8 µM (derivative **76**). Another chemical modification explored was the benzylic oxidation of ester derivative **83**, which increased the anticancer activity, as evidenced by a decrease in the IC_50_ from 26 to 15.1 µM [41]. The most active derivatives (**75**–**77**, **81**, and **84**) consistently exhibited enhanced antiproliferative activity across a range of cancer cell lines, including colorectal, melanoma, and pancreatic cells, surpassing the efficacy of CA **1** (Table 11) [40,41].

Further studies have explored the mechanisms underlying the antiproliferative effects of the most active derivatives (**76** and **81**) in SW480 cells [40,41]. Both compounds demonstrated selectivity for cancer cells over normal cells, suggesting a favorable therapeutic index. These compounds modulated cell cycle progression and oxidative stress in SW480 cells. Compound **76** induced G0/G1 phase arrest by reducing CDK4 and CDK6 levels and exhibited antioxidant effects. Compound **81** initially showed a similar effect, but upon prolonged exposure, it promoted pro-oxidant effects and caused a shift in cell cycle arrest to the S phase. Molecular docking studies demonstrated favorable interactions between both compounds and the active site of CDK6, suggesting that CDK6 is a potential molecular target.

## 6. SAR Considerations

**Figure 12:**
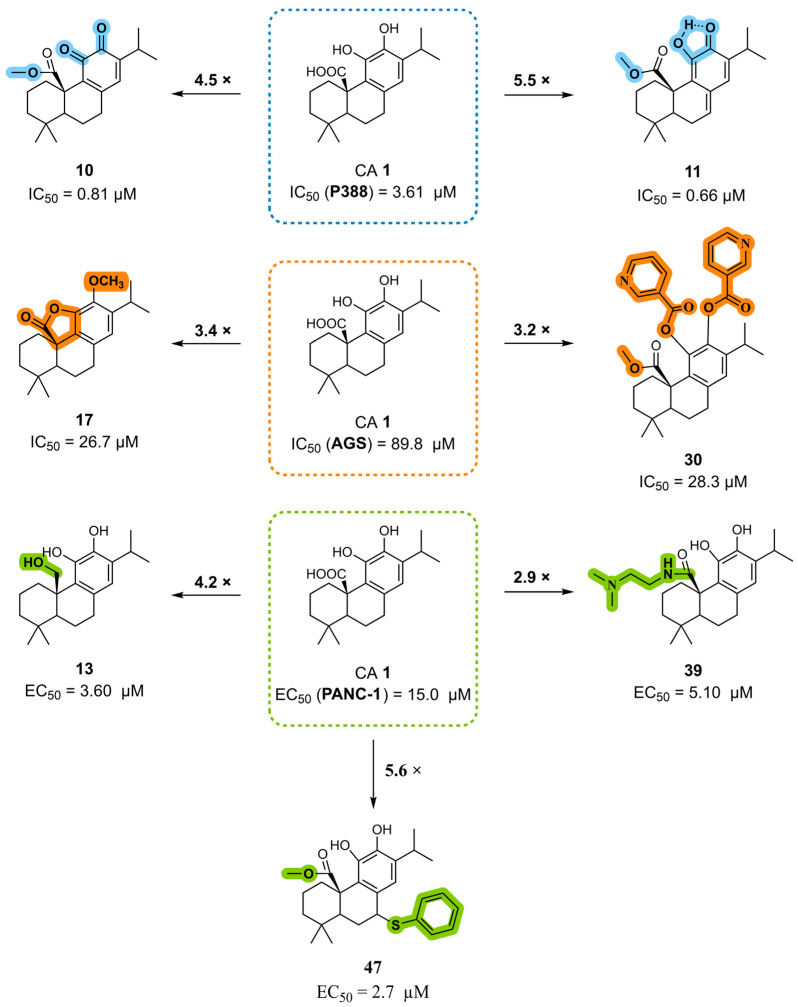
The main SAR conclusions derived from the antiproliferative activity of the CA **1** derivatives in different cancer cell lines (P388, AGS, and PANC-1).

**Figure 13:**
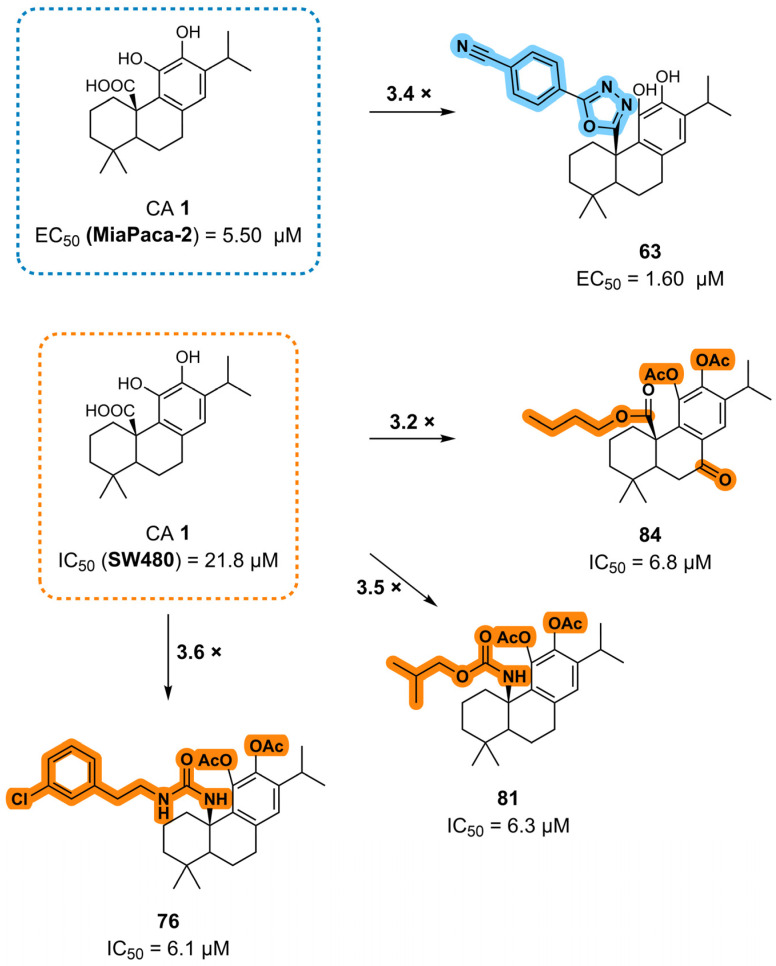
The main SAR conclusions derived from the antiproliferative activity of the CA **1** derivatives in MiaPaca-2 and SW480 cancer cell lines.

The chemical modifications performed on the CA **1** backbone include modifications of the hydroxyl groups at C11 and C12, the carboxylic acid at C20, and the benzylic position at C7, leading to the generation of several derivatives with markedly improved anticancer activity compared to the parent compound (CA **1**). These results underscore the chemical versatility of the CA scaffold. The main SAR considerations are illustrated in Figure 12 and Figure 13, demonstrating that targeted modifications at specific positions are crucial for enhancing antiproliferative activity.

The combination of esterification at C20 with the introduction of an o-quinone or o-quinone equivalent in the C-ring markedly enhances the anticancer activity of CA **1**. However, the impact of this structural modification appears to depend on the cancer cell line. While derivatives such as compounds **10** and **11** show notably increased potency in P388 murine leukemia cells, similar quinone-containing structures exhibit a loss of activity in pancreatic cancer cells, indicating that their biological performance varies substantially across different cancer types.

Additionally, methylation at the C12 position, combined with the introduction of a γ-lactone ring, as in compound **17**, increased the anticancer activity. Esterification of the hydroxyl groups at C11 and C12, namely the incorporation of a nicotinate moiety, along with a methyl ester at C20, also enhanced potency, as exemplified by compound **30**.

Chemical modifications at the C20 position are among the most effective strategies for improving activity, leading to the development of several highly potent derivatives. The introduction of an amide group, especially a dimethylaminoethyl substitution (compound **39**), was advantageous. The reduction of the carboxylic acid (C20) to its corresponding alcohol, as observed for derivative **13**, resulted in increased activity. The incorporation of heterocyclic elements, such as a 1,3,4-oxadiazole bearing a phenyl substituent, also contributed to enhanced potency. Furthermore, the introduction of urea, carbamate, or ester moieties at C20 consistently improved the antiproliferative activity. Within the urea and carbamate series, longer aliphatic chains correlated with lower IC_50_ values, suggesting that hydrophobic chain elongation enhances biological performance. Among the carbamates, compound **81**, which features a 2-methylpropyl substituent, exhibited the highest activity. In urea derivatives, the insertion of electronegative atoms and aromatic rings into the urea side chain further improved the activity, with compound **76**, which bears a chlorine-substituted benzene ring, emerging as the most potent example.

Combinations of modifications at multiple positions can result in further activity improvement. For instance, compound **47**, which combines esterification at C20 and a benzene ring at the C7 benzylic position via a sulfur linker, exhibited superior activity. Similarly, compound **84**, featuring a butyl ester at C20 in combination with a carbonyl group at C7, demonstrated improved anticancer properties.

In summary, the SAR analysis of CA **1** semisynthetic derivatives identified the introduction of amide, ester, urea, and carbamate groups at the C20 position as a key strategy for enhancing anticancer activity, with consistently improved antiproliferative effects relative to the parent scaffold. Activity gains were most pronounced when these functional groups incorporated aromatic moieties, electronegative atoms, or longer aliphatic chains, suggesting that increased hydrophobic interactions and/or favorable electronic effects play a central role in modulating biological activity. Modifications at the benzylic position (C7) also enhanced the activity when combined with favorable C20 substitutions, indicating a cooperative effect between these two positions in enhancing anticancer activity. In contrast, the introduction of C-ring quinone resulted in variable antiproliferative effects depending on the cancer cell line evaluated, highlighting the limitations of this modification. These results highlight the chemical versatility of the CA **1** scaffold and demonstrate that targeted modifications are central to enhancing antiproliferative effects.

## 7. Conclusions and Future Perspectives

CA **1** has emerged as a promising scaffold for anticancer drug development owing to its ability to modulate multiple key cellular pathways associated with tumor initiation, progression, and dissemination. Across diverse cancer models, CA **1** has been shown to inhibit proliferation; induce cell death through distinct pathways; arrest the cell cycle; suppress metastasis and angiogenesis; and modulate oxidative stress, inflammation, and cellular metabolism. Its ability to attenuate multidrug resistance further reinforces its potential value. Taken together, these findings highlight the therapeutic relevance of CA **1** in cancer treatment.

Although CA **1** exerts multifaceted anticancer action, its anticancer potency remains modest, motivating the development of semisynthetic derivatives with improved efficacy. Some of these derivatives exhibited enhanced potency compared with the parent compound, achieving promising results and serving as valuable lead compounds for further optimization. However, some important challenges remain. Many derivatives still show only moderate antiproliferative activity, and their selectivity toward cancer cells often remains insufficient, with some compounds displaying comparable effects in normal cells. This highlights the need for chemical modifications of the CA **1** scaffold to generate derivatives with higher potency and improved selectivity, thereby increasing their therapeutic potential.

Despite these advances, several limitations associated with the available literature should be acknowledged. Most of the reported anticancer effects of CA **1** and its semisynthetic derivatives are based on in vitro studies, frequently employing different cancer cell lines, experimental conditions, and assay methodologies, which limits a direct comparison across studies. In addition, in vivo validation, pharmacokinetic characterization, and toxicity assessments remain scarce, limiting conclusions regarding translational potential.

Future studies on the most promising semisynthetic derivatives should include the evaluation of pharmacokinetic properties, metabolic stability, and toxicity in relevant preclinical models, as well as the development of advanced delivery strategies to enhance bioavailability and promote efficient tumor targeting. Collectively, these efforts will be crucial to fully exploiting the therapeutic potential of CA **1** and its semisynthetic derivatives.

## Figures and Tables

**Table 1:** Inhibitory activity of CA **1** on the proliferation of different human cancer cell lines.

Type of Cancer	Cell Line	IC_50_ (µM) ^1^	Time (h)	Assay	Ref.
**Brain**	PT2	27.5	48	MTT	[48]
**Breast**	MCF-7	47.5	24	MTT	[49]
	MDA-MB-231	29.3	24	MTT	[49]
**Colorectal**	SW480	28.2	72	CCK-8	[50]
	HCT116	32.52	72	CCK-8	[50]
		26.80	24	MTS	[51]
		27.67	48	MTS	[51]
		28.95	72	MTS	[51]
	HT-29	30.13	72	CCK-8	[50]
		48.5	24	CellTiter 96 Aqueous Non-Radioactive Cell Proliferation	[52]
	Caco-2	92.1	24	CellTiter 96 Aqueous Non-Radioactive Cell Proliferation	[52]
		23	24	Thymidine incorporation	[53]
	LoVo	26.4	24	CellTiter 96 Aqueous Non-Radioactive Cell Proliferation	[52]
	COLO 205	32.8	24	MTT	[54]
**Gastric**	AGS	59.86	24	MTT	[55]
		56.94	48	MTT	[55]
		49.84	72	MTT	[55]
	MKN-45	72.07	24	MTT	[55]
		61.33	48	MTT	[55]
		53.42	72	MTT	[55]
**Leukemia**	CML KBM-7	25	48	MTT	[56]
	HL-60	1.7	24	MTT	[54]
	CEM/ADR5000	11.48	72	Resazurin	[57]
	CCRF-CEM	19.50	72	Resazurin	[57]
**Liver**	HepG2	43.7	24	MTT	[58]
	SMMC-7721	74.8	24	MTT	[58]
**Lung**	H1299	47.3	24	Crystal violet	[59]
		27.1	48	Crystal violet	[59]
	H460	89.6	24	Crystal violet	[59]
		67	48	Crystal violet	[59]
	A549	42.42	72	CCK-8	[50]
		12.5	-	CCK-8	[60]
**Neuroblastoma**	IMR-32	30	24	MTT	[61]
**Oral**	CAL27	34.71	24	CCK-8	[62]
	SCC9	32.73	24	CCK-8	[62]
**Prostate**	PC-3	64	-	Crystal violet	[63]
		41.1	48	MTT	[64]
	LNCaP	21	-	Crystal violet	[63]
**Skin**	A375	22.74	48	MTT	[65]
	MelJuSo	35.34	48	MTT	[65]
	B16F10	7.08	24	MTT	[66]

^1^ IC_50_ is the concentration of the compound required to inhibit 50% of cell growth.

**Table 2:** In vitro anticancer activity of CA **1** against brain, breast, and cervical cancers.

Type of Cancer	Cell Line	Effects on Targeted Cells	Molecular Pathways/Targets	Ref.
**Brain**	PT2	Antiproliferation Apoptosis Cell cycle arrest (early G2)	↑ p21^WAF^, p-AKT	[48]
			↓ cyclin B1, p-STAT3, Rb, p-Rb, SOX2, GFAP, CDK’s activity	
**Breast**	184-B5/HER	Antiproliferation Apoptosis Cell cycle arrest (G2/M phase)	↑ cyclin B1	[67]
	MCF-7 MDA-MB-231	Antiproliferation Apoptosis	↑ caspases-3/7 activity, ROS	[49]
**Cervical**	CaSki	Antiproliferation Apoptosis Cell cycle arrest (G2/M) Inhibition of migration	↑ ROS, p-JNK, Bax, Bak, Bad, Apaf1, c-caspases-3/9, cytoplasm cytochrome c, p-ERK1/2, p-p38, ATF2, ATF4, XBP1, CREB, caspases-3/9 activity	[68]
			↓ Bcl-2	
	SiHA	Antiproliferation Apoptosis	↑ caspases-3/9 activity	[68]

**Table 6:** Cell viability (IC_50_) of the CA **1** and derivatives **16**–**27** against gastric, liver and lung cancer cell lines, as well as normal lung fibroblasts.

Compound	IC_50_ (µM)				Ref.
	Gastric	Liver	Lung	Non-Tumoral	
	AGS	HepG2	SK-MES-1	MRC-5	
**CA 1** ^1^	89.8	41.3	N.D.	21.8	[36]
**16** ^1^	45.2	43.5	N.D.	54.9	[36]
**17** ^1^	26.7	58.1	N.D.	31.9	[36]
**18** ^1^	246	240	N.D.	293	[36]
**19** ^1^	103	203	N.D.	130	[36]
**20** ^1^	99.5	56.1	N.D.	44.4	[36]
**21** ^1^	133	522	N.D.	516	[36]
**22** ^1^	85.6	251	N.D.	109	[36]
**23** ^1^	46.8	120	N.D.	54.5	[36]
**24** ^2^	39.2	N.D.	81.7	45.1	[37]
**25** ^2^	41.0	N.D.	76.0	46.9	[37]
**26** ^2^	48.9	N.D.	73.0	43.4	[37]
**27** ^2^	>100	N.D.	>100	>100	[37]

N.D.: not determined. ^1^ IC_50_ values were determined using the neutral red uptake (NRU) assay after 24 h of incubation with increasing concentrations of each compound. ^2^ IC_50_ values were determined using the MTT assay after 72 h of incubation with increasing concentrations of each compound.

**Table 7:** Cell viability (IC_50_) of the CA **1** and derivatives **12**, **28–33** against gastric and liver cancer cell lines, as well as normal lung fibroblasts [38].

Compound	IC_50_ (µM)		
	Gastric	Liver	Non-Tumoral
	AGS	HepG2	MRC-5
**CA 1**	89.8	41.3	21.8
**12**	48.4	46.6	18.3
**28**	17.7	31.2	15.4
**29**	47.9	203.3	21.5
**30**	28.3	23.3	51.5
**31**	40.1	43.9	77.8
**32**	90.3	27.7	33.4
**33**	52.7	91.4	86.1

IC_50_ values were determined using the neutral red uptake (NRU) assay after 24 h of incubation with increasing concentrations of each compound.

**Table 8:** IC_50_ values of compounds on HsFPPS activity [39].

Compound	HsFPPS	Compound	HsFPPS	Compound	HsFPPS
	IC_50_ (µM)		IC_50_ (µM)		IC_50_ (µM)
**CA 1**	20.0	**44**	3.10	**58**	2.20
**12**	0.865	**45**	0.800	**59**	0.859
**13**	7.80	**46**	0.520	**60**	1.0
**18**	10.6	**47**	28.0	**61**	0.644
**34**	0.833	**48**	1.30	**62**	0.194
**35**	>100	**49**	1.10	**63**	7.40
**36**	41.2	**50**	13.3	**64**	>100
**37**	2.70	**51**	0.389	**65**	12.5
**38**	6.03	**52**	0.523	**66**	>100
**39**	1.20	**53**	0.234	**67**	0.335
**40**	0.914	**54**	18.7	**68**	0.252
**41**	2.70	**55**	0.596	**69**	0.289
**42**	35.0	**56**	13.3	**Zoledronate**	0.100–0.200
**43**	1.50	**57**	0.473		

**Table 9:** Cell viability (EC_50_) of the compounds against two pancreatic cancer cell lines (PANC-1 and MiaPaca-2) [39].

Compound	EC_50_ (µM) ^1^		Compound	EC_50_ (µM) ^1^	
	PANC-1	MiaPaCa-2		PANC-1	MiaPaca-2
**CA 1**	15.0	5.50	**51**	>50.0	>50
**12**	12.0	13.0	**52**	>50.0	>50
**13**	3.60	>50.0	**54**	>50.0	>50.0
**18**	>100	>100	**55**	21.2	19.3
**34**	8.00	>100	**57**	40.6	32.5
**36**	31.7	46.4	**59**	>50.0	N.D.
**39**	5.10	9.20	**60**	12.3	14.0
**41**	>50.0	38.6	**61**	8.10	13.2
**42**	24.0	16.5	**62**	18.3	42.2
**44**	>50.0	11.4	**63**	5.90	1.60
**45**	48.0	29.6	**65**	7.00	11.6
**46**	10.0	>100	**67**	50.0	N.D.
**47**	2.7	11.1	**68**	13.0	29.4
**48**	12.9	12.2	**69**	45.0	>100
**49**	13.3	8.20	**Zoledronate**	16.1–24.2	13.4

EC_50_ values were determined using the MTT assay after 72 h of incubation with increasing concentrations of each compound. N.D.: not determined. ^1^ EC_50_ is the concentration of the compound required to produce 50% of its maximum effect.

**Table 10:** Cell viability (IC_50_) of the CA **1** and derivatives **35**, **70**–**84** against the CRC cell line.

Compound	IC_50_ (µM)	Reference
	Colorectal	
	HCT116	
CA **1**	42	[40,41]
**35**	28	[41]
**70**	55	[40]
**71**	44	[40]
**72**	31	[40]
**73**	22	[40]
**74**	41	[40]
**75**	14	[40]
**76**	9.8	[40]
**77**	12	[40]
**78**	21	[40]
**79**	55	[41]
**80**	37	[41]
**81**	17	[41]
**82**	25	[41]
**83**	26	[41]
**84**	15.1	[41]

IC_50_ values were determined using the MTT assay after 72 h of incubation with increasing concentrations of each compound.

**Table 11:** Cell viability (IC_50_) of CA **1** and derivatives **75**–**77**, **81**, and **84** against colorectal, melanoma, and pancreatic cancer cell lines, as well as a normal fibroblast cell line.

Compound	IC_50_ (µM)						Reference
	Colorectal			Melanoma	Pancreas	Non-Tumoral	
	SW480	SW620	Caco-2	A375	MiaPaca-2	BJ	
**CA 1**	21.8	18	34	27.6	21	N.D.	[40,41]
**75**	8.6	9	10.9	N.D.	N.D.	N.D.	[40]
**76**	6.1	6.1	8.0	7.0	9.0	>25	[40]
**77**	7.6	6.4	9.6	7.6	9.3	N.D.	[40]
**81**	6.3	10	16	7.5	6.7	>50	[41]
**84**	6.8	11	12.7	12	8.1	N.D.	[41]

IC_50_ values were determined using the MTT assay after 72 h of incubation with increasing concentrations of each compound. N.D.: not determined.

## Data Availability

No new data were created or analyzed in this study. Data sharing is not applicable to this article.
